# Three-Dimensional Electro-Anatomical Mapping and Myocardial Work Performance during Spontaneous Rhythm, His Bundle Pacing and Right Ventricular Pacing: The EMPATHY Study

**DOI:** 10.3390/jcdd9110377

**Published:** 2022-11-02

**Authors:** Michele Malagù, Francesco Vitali, Rodolfo Francesco Massafra, Laura Sofia Cardelli, Rita Pavasini, Gabriele Guardigli, Claudio Rapezzi, Matteo Bertini

**Affiliations:** Cardiology Unit, Azienda Ospedaliero-Universitaria di Ferrara, Via Aldo Moro 8, 44124 Ferrara, Italy

**Keywords:** pacemaker, physiology, electrophysiology, electromechanical, speckle tracking, strain

## Abstract

Background. His bundle pacing (HBP) has emerged as an alternative site to right ventricular pacing (RVP) with encouraging outcomes. To date, no study has investigated the systematic approach of three-dimensional electroanatomic mapping (3D-EAM) to guide HBP implantation and to evaluate myocardial activation timing. Furthermore, studies reporting a comprehensive assessment of the ventricular function, using myocardial work (MW) evaluation are lacking. Objectives. (1) To evaluate the systematic use of the 3D-EAM as a guide to HBP; (2) to assess the electrical and mechanical activations with high-density mapping, comparing spontaneous ventricular activation (SVA), HBP and RVP; (3) to assess the myocardial function through speckle-tracking echocardiography (STE) and MW analysis in SVA, HBP and RVP. Methods. 3D-EAM was performed in consecutive patients undergoing HBP implantation with a low use of fluoroscopy. All patients were systematically evaluated with high-density mapping, MW and STE. Results. Fifteen patients were enrolled, of whom three had an implant failure (20%). RV activation time was not statistically different between SVA and HBP (103 vs. 104 ms, *p* = 0.969) but was significantly higher in RVP (133 ms, *p* = 0.011 vs. SVA and *p* = 0.001 vs HBP). Global constructive work was significantly lower during RVP (1191 mmHg%) than during SVA and HBP (1648 and 1505 mmHg%, *p* = 0.011 and *p* = 0.008, respectively) and did not differ between SVA and HBP (*p* = 0.075). Conclusions. 3D-EAM and MW evaluation showed that HBP was comparable to the physiological SVA in terms of activation time and cardiac performance. Compared to both SVA and HBP, RVP was associated with a worse activation timing and ventricular efficiency.

## 1. Introduction

The harmful effect of right ventricular pacing (RVP), both at mid- and long-term follow-up, lead to a continuous search for alternative pacing sites, among which His bundle pacing (HBP) emerged, showing encouraging results [1]. HBP ideally represents the most physiological approach to ventricular stimulation, allowing the electrical activation through the normal pathway of the His–Purkinje system. Unlike RVP, HBP has been hypothesized to result in asynchronous ventricular activation, a lower interventricular dyssynchrony and a reduced dispersion of ventricular repolarization [2].

Several small observational studies have been published in the last 20 years, suggesting the interesting potential for HBP. In detail, when compared to apical pacing, HBP showed less ventricular dyssynchrony, less mitral regurgitation and a better left ventricular systolic function at standard and tissue Doppler echocardiography [3,4,5,6]. The advantages of HBP versus RVP have also been proven in terms of a better quality of life, NYHA class, 6-min walking distance and cardiopulmonary reserve [7,8]. Although data come from small non-randomized non-controlled studies, HBP is also associated with a reduction in death and heart failure hospitalization events at long term follow-up [9]. However, a major limitation of this technique is the high failure rate, with implant success rate varying from 72% to 88% [8,9,10]. The electrical parameters in the His area may be poor and HBP requires more revisions and generator changes during follow-up [9].

To date, no study has described the role of three-dimensional electro-anatomical mapping (3D-EAM) as a guide to HBP system implantation and in the evaluation of ventricular function. Furthermore, no study has provided a comprehensive assessment of ventricular function using contemporary echocardiographic functional imaging, namely changes in myocardial work parameters according to HBP stimulation.

The aims of this study were: (i) to report on success rate and electrical parameters of HBP performed with systematic use of 3D-EAM, (ii) to evaluate electrical and mechanical ventricular activations comparing HBP, spontaneous rhythm and RVP by means of high-density mapping, (iii) to assess myocardial function through speckle-tracking echocardiography and myocardial work analysis.

## 2. Methods

The Electrical and Mechanical activation in PAcing The His bundle conduction sYstem (EMPATHY) study is a prospective, single center, cohort study, enrolling consecutive patients undergoing HBP at the Cardiology Unit of Azienda Ospedaliero-Universitaria di Ferrara, Italy. The study was registered on ClinicalTrials.gov (NCT05222672). The protocol was approved by the local ethics committee and informed consent was signed by all patients.

The inclusion criteria were: (1) class I or IIa indication for pacemaker implantation, according to European guidelines [11]; (2) age ≥18 years; (3) signed written informed consent. The exclusion criteria were: (1) inability to express informed consent; (2) pregnancy; (3) severe mitral or aortic valve disease; (4) left ventricular ejection fraction (LVEF) <35%.

The primary endpoint of the study was to assess the success rate of HBP using 3D-EAM and to evaluate electrical and mechanical activation of ventricular myocardium and ventricular performance assessed with 3D-EAM and myocardial work (MW) during HBP, RVP and spontaneous rhythm.

### 2.1. Implantation

The index procedure was comprehensive of pacemaker implantation and simultaneous 3D-EAM. The right ventricle and the His bundle area were non-fluoroscopically mapped with a high-density catheter, inserted via the femoral vein. The pacing leads were inserted via the left cephalic or axillary vein, mapped in the 3D-EAM and inserted with the use of 3D-EAM and fluoroscopy. For His bundle pacing, an active fixation lead (SelectSecure 3830, Medtronic, Minneapolis, MN, USA or Solia S, Biotronik, Berlin, Germany) was positioned, using a non-deflectable sheath (C315, Medtronic, Minneapolis, MN, USA or Selectra 3D, Biotronik, Berlin, Germany). The position of the His bundle lead was confirmed with unipolar and bipolar intracardiac electrograms. Standard criteria were used to define selective and non-selective His capture [12]. A backup right ventricular lead was implanted in all patients. An atrial lead was implanted if needed, according to the pacing indication. Antibiotic prophylaxis and antithrombotic drugs were managed according to Center protocols [13,14].

### 2.2. Imaging

High density 3D-EAM of the right ventricle was performed via the femoral vein at the time of pacemaker implantation, using Advisor HD Grid catheter and EnSite Precision mapping system (Abbott, Chicago, IL, USA) [15]. At least 2000 points were required for each map. The right ventricle activation time was calculated from the earliest to the latest activation in the mapping system.

Following the procedure, all patients underwent an echocardiographic evaluation. Basic information, such as left ventricular (LV) volume (mL), LVEF (%), atrial volume (mL) and degree of valve diseases, were collected. Global longitudinal strain (GLS) and MW were analyzed. MW is a novel echocardiographic technique, based on the speckle tracking analysis, which estimates the left ventricular performance by area under pressure-strain loops curve, derived non-invasively from GLS and blood pressure [16,17,18]. All echocardiographic examinations were performed with GE Vivid E9 with M5S transducers, GLS and myocardial work analyses were post-processed offline with EchoPAC software V.202 (GE Healthcare, Chicago, IL, USA). The echocardiographic parameters were assessed according to international standards [19]. The collected MW parameters were: (i) global constructive work (GCW), defined as the arithmetic sum of the work performed during myocardial shortening in systole and myocardial lengthening during isovolumetric diastole; (ii) global wasted work (GWW), the arithmetic sum of the work performed by myocardial lengthening in systole and myocardial shortening during isovolumetric diastole; (iii) global work index (GWI), the work performed in the entire systole, namely the work between mitral valve closure and opening; (iv) global work efficiency (GWE), expressed as the percentual ratio between the GCW and the sum of GCW and GWW.

A twelve-leads ECG was performed and analyzed.

3D-EAM, echocardiography and ECG evaluations were performed in each of the following conditions: spontaneous ventricular activation (SVA), HBP and RVP. HBP and RVP measurements were performed during pacing at a fixed rate in DDD mode with an optimized AV delay or in VVI mode, depending on whether patients were in sinus rhythm or atrial fibrillation. 

### 2.3. Statistics

Continuous variables were expressed as mean ± standard deviation when normally distributed, as estimated using the Shapiro–Wilk test, or as median and interquartile range. Categorical variables were expressed as percentages. Differences between repeated measurements during SVA, HBP and RVP, were assessed using the ANOVA test or the Wilcoxon rank-sum test, respectively, for normally and non-normally distributed variables. Parametric and non-parametric multiple comparisons were tested with the Tukey and Bonferroni methods. *p* values < 0.05 were considered statistically significant. The statistical analysis was performed using SPSS Statistics, version 25.0 (IBM, Armonk, NY, USA).

## 3. Results

Fifteen patients were enrolled in the study. The mean age was 76 ± 12 years and 13 were male (86%). The baseline clinical characteristics are summarized in Table 1.

Procedural data are shown in Table 2. Procedural success was reached in 80% of the patients (12/15). Causes for implant failure were inability to obtain HBP due to a high threshold (>5 V at 1.0 ms) in one patient and lead instability with dislocation during implantation in the other two patients. His capture was selective in five patients.

3D-EAM, ECG and MW data are reported in Table 3. A mean of 4783 ± 3728 map points was obtained in a time of 343 ± 186 sec. The right ventricular median activation time was not statistically different between SVA and HBP (103 (92–140) vs. 104 (95–108) ms, *p* = 0.969,) but was significantly higher in RVP (133 (120–147) ms, *p* = 0.011 vs. SVA and *p* = 0.001 vs. HBP). The QRS duration was significantly higher in RVP (168 ± 23 ms) than in SVA (120 ± 31 ms, *p* = 0.002) and HBP (123 ± 24 ms, *p* = 0.002) but did not differ between SVA and HBP (*p* = 0.929).

**Figure 1:**
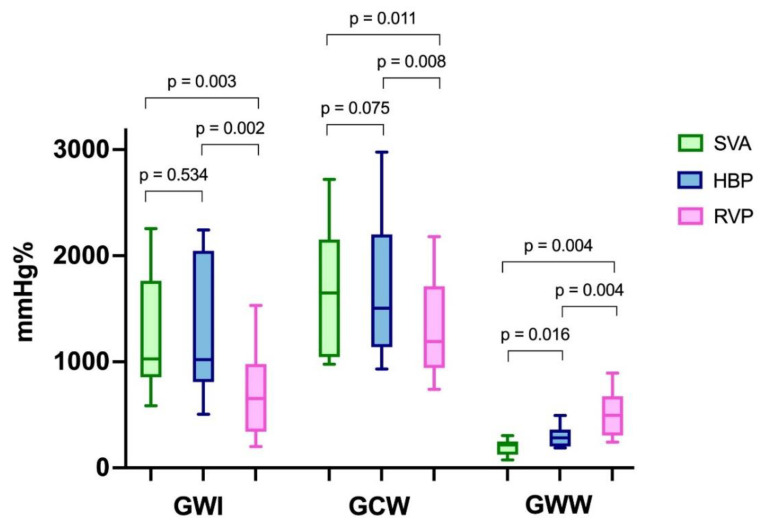
Myocardial work. GWI: global work index. GCW: global constructive work. GWW: global wasted work. SVA: spontaneous ventricular activation. HBP: His bundle pacing. RVP: right ventricular pacing.

**Figure 2:**
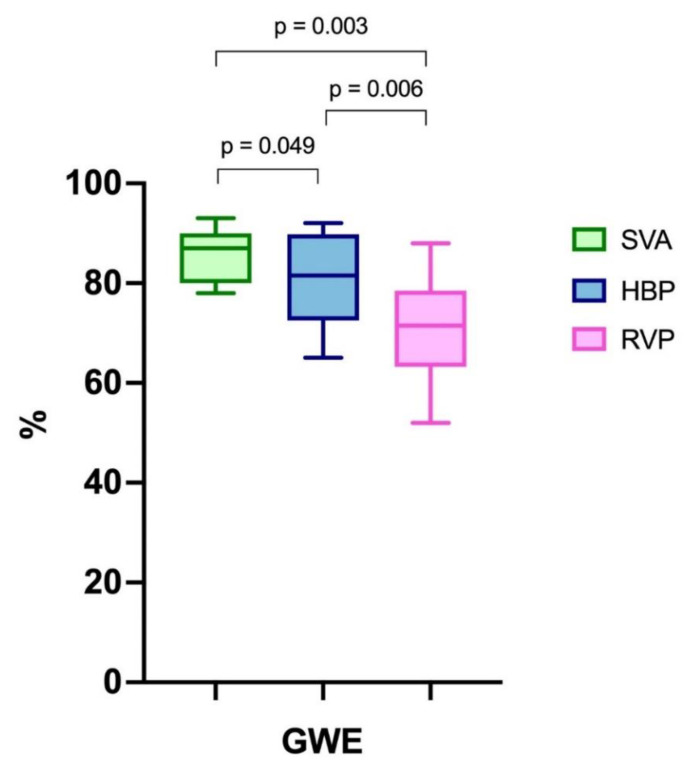
Myocardial work. GWE: global work efficiency. SVA: spontaneous ventricular activation. HBP: His bundle pacing. RVP: right ventricular pacing.

**Figure 3:**
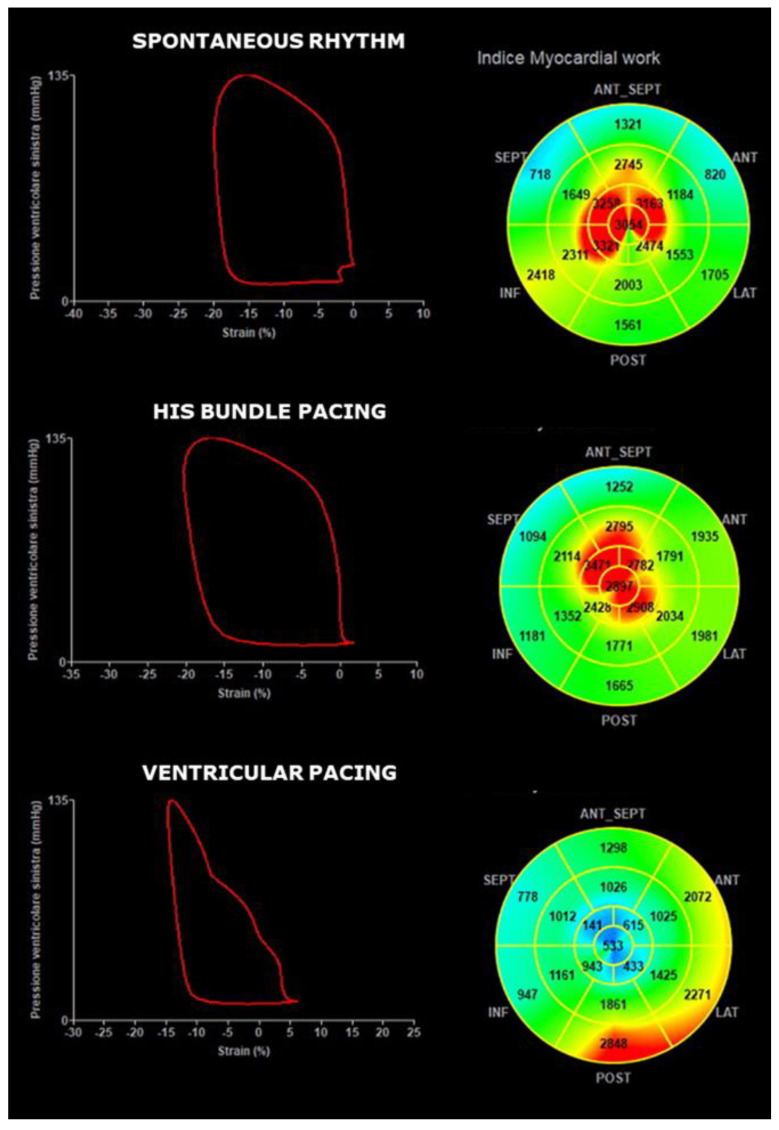
Pressure strain loops and global longitudinal strain bulls-eye showing the difference in left ventricular activation between spontaneous rhythms, His bundle pacing and right ventricular pacing.

GLS, GWI and GCW were all significantly higher during SVA or HBP than during RVP and did not differ between SVA and HBP (Figure 1). The GWW was significantly different between the three groups, with the higher values with RVP stimulation and the lower with SVA. GWE was slightly lower in HBP compared to SVA, but GWE was strongly and significantly lower with RVP compared to both SVA and HBP (Figure 2 and central illustration). A representation of the pressure strain loops and the GLS bulls-eye is shown in Figure 3.

At the one month follow-up, one patient died by a non-cardiac cause. The electrical parameters of the surviving patients are reported in Table 4.

## 4. Discussion

**Figure 4:**
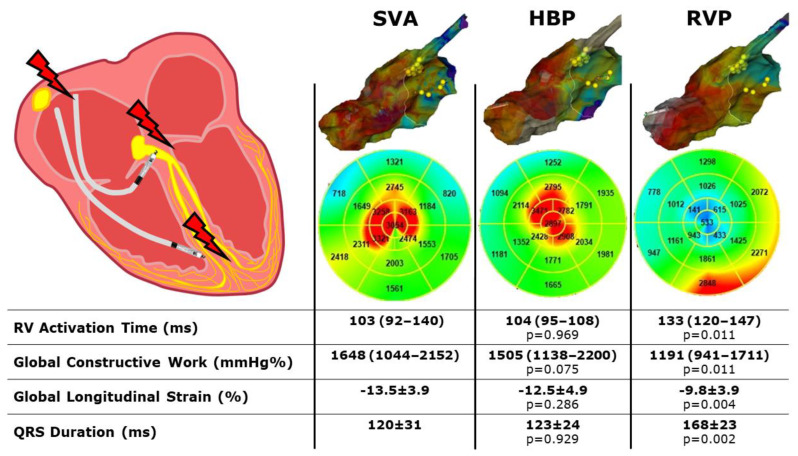
Central illustration. Three dimensional high-density electroanatomical mapping and myocardial work performance during spontaneous rhythm, His bundle pacing and right ventricular pacing. SVA: spontaneous ventricular activation; HBP: His bundle pacing; RVP: right ventricular pacing; RV: right ventricle. *p* values are considered vs SVA.

The main finding of the current study is that HBP implantation guided by the 3D-EAM leads to right ventricular activation time and myocardial performance similar to spontaneous rhythm and definitely better than RVP (Figure 4: Central illustration). Moreover, HBP was feasible in 80% of the study population with a low utilization of fluoroscopy. 

### 4.1. Procedural Success

The procedural failure of HBP implantation in our cohort was 20% (3/15 patients). Previous studies reported rates of implant failure ranging between 12%–28% [8,9,10]. Therefore, our results are consistent with the literature. This means that 3D-EAM, which may be important to reduce fluoroscopy time during the HBP implantation, seems to not increase the procedural success rate, indicating, once again, how important are the anatomic barriers in this complex procedure [20]. In case of HBP failure, left bundle branch pacing has been proposed as an effective alternative with a good success rate and satisfying electrical parameters [21]. We usually perform left bundle branch pacing in patients in whom HBP failed, and we performed it in the three patients with implant failure. However, the evaluation of left bundle branch pacing goes beyond the purpose of the present study and those patients were excluded from the analysis.

### 4.2. Three-Dimensional Electro-Anatomic Mapping

Our study showed that right ventricular activation time, assessed with 3D-EAM, is not different between SVA and HBP but is significantly lower in both these stimulation modalities, compared to the conventional RVP. This result is consistent with the hypothesis that HBP provides a “physiological” electrical activation of the ventricular myocardium [1]. Previous studies showed that RVP was associated with a lower left ventricular ejection fraction, a higher interventricular delay and a higher asynchrony index, assessed with standard and tissue Doppler echocardiography, compared to HBP [4]. Our study provided the first systematic assessment of ventricular activation by means of a high-density 3D-EAM. The advantages of the 3D-EAM are that the whole cardiac chamber is directly mapped by dragging a catheter over the endocardial surface, recording the electrical signals and providing a precise measure of the activation time. This finding could explain, from an electrophysiological basis, why patients with RVP have a significantly higher rate of pacing-induced cardiomyopathy than patients with HBP [9]. Furthermore, our data showed no difference between the physiological SVA and the artificial HBP.

### 4.3. Myocardial Work

For all the MW indexes considered, RVP resulted in worse performance, compared to SVA. Conversely, HBP was non different to SVA in terms of GWI and GCW. GWW and GWE were slightly lower, compared to SVA and HBP. 

Those findings indicate a worse left ventricular efficiency associated with RVP, in whom a major quote of myocardial energy is wasted. HBP had a lower impact over the myocardial efficiency, with GWI and GCW comparable to the physiological left ventricular contraction. However, GWW resulted higher in HBP than in SVA (even if not as much higher as during RVP), driving a slightly worse GWE. This might also be due to the small sample size, considering the small numerical difference between the two groups which instead becomes considerably consistent, in comparison to RVP, to the detriment of the latter.

The echocardiographic MW is a function of pressure and strain, so it is not a direct measure of work but rather a surrogate index, while the invasive MW is expressed as pressure over volume, ultimately equivalent to the force times length. However, the echocardiographic MW is a robust index, validated against the invasive MW, of valuable significance in various clinical conditions, such as myocardial infarction, arterial hypertension, heart failure, cardiac resynchronization therapy and left bundle branch area pacing [22,23,24,25].

### 4.4. ECG

It is the basis of the physiological pacing that QRS duration and morphology during HBP are nearly identical to spontaneous ventricular rhythm, particularly in case of selective His capture [2]. Previous studies reported that the native QRS duration was not different from the His-paced QRS duration [3]. Our study provides a further confirmation of previous findings.

### 4.5. Follow-Up

We observed an increase in HBP threshold at one month follow-up. Previous studies showed a significant increase in the His bundle capture threshold at 5-year follow-up, compared to implant measurements [9]. Our study was not designed to collect data over a follow-up longer than one month but our results are in line with the literature.

## 5. Limitations

Our cohort is relatively small, with only 15 patients, of whom 12 were successfully implanted. However, those numbers resulted sufficient to show a statistical significance. Previous studies exploring the hemodynamic and the performance of HBP enrolled comparable numbers of patients [3,4,5,7,8].

Only the RV activation time, and not the biventricular activation time, was collected. This limitation could not be overcome, since left ventricular mapping would have been adding a procedural risk for the patients, but having the activation time of the cardiac chamber where stimulation starts, combined with the indexes of the myocardial performance, is a good surrogate to explore the acute effects of physiologic stimulation.

## 6. Conclusions

Electrophysiologic study with 3D-EAM and echocardiographic MW evaluation showed that HBP is comparable to physiological SVA, in terms of activation time and cardiac performance. Compared to both SVA and HBP, RVP was associated with worse right ventricular activation time and left ventricular efficiency.

## Figures and Tables

**Table 1:** Baseline clinical characteristics.

	Overall Population *n =* 15
Age (years)	76 ± 12
Weight (kg)	79 ± 15
BMI (kg/m^2^)	26 ± 3
BSA (m^2^)	1.9 ± 0.4
Coronary Artery Disease	26.7%
Heart Failure	33.3%
Atrial Fibrillation	53.3%
Diabetes	0%
Hypertension	80%
Dyslipidemia	53.3%
Smoke history	46.7%
COPD	13.3%
Cancer history	40%
Hemoglobin (g/dL)	13.0 ± 1.4
eGFR, Cockroft–Gault (mL/min)	66 ± 30
ACE inhibitors	53.3%
Beta blockers	53.3%
Anticoagulants	60%
Antiplatelets	40%
Ejection fraction (%)	51 ± 14
LV EDVi (mL/m^2^)	60 (34–78)
LAVi (mL/m^2^)	41 ± 10
RAVi (mL/m^2^)	35 ± 13

BMI: body mass index. BSA: body surface area. COPD: chronic obstructive pulmonary disease. eGFR: estimated glomerular filtration rate. LV EDVi: left ventricular end diastolic volume index. LAVi: left atrial volume index. RAVi: right atrial volume index.

**Table 2:** Procedural data.

	Overall Population *n* = 15
Pacing Indication	
AV block	46.7%
AF with a slow ventricular conduction	26.7%
AF undergoing an AV nodal ablation (pace & ablate)	26.7%
Atrial lead implanted	46.7%
Axillary venous access for the His lead	100%
Procedure duration (min)	130 (120–157)
Fluoroscopy time (sec)	780 (529–789)
DAP (µGym^2^)	4442 ± 2981
HB lead sensing (mV, *n* = 12)	2.3 (1.6–4.6)
RV lead sensing (mV)	9.0 (7.9–16.8)
HB lead impedance (Ohm, *n* = 12)	491 ± 107
RV lead impedance (Ohm)	639 ± 111
HB lead pacing threshold at 0.4 ms (V, *n* = 12)	0.7 (0.4–2.6)
HB lead pacing threshold at 1.0 ms (V, *n* = 12)	0.7 (0.4–1.5)
RV lead pacing threshold at 0.4 ms (V)	0.5 (0.5–0.6)

DAP: dose area product. AV: atrio-ventricular. AF: atrial fibrillation. HB: His bundle. RV: right ventricle.

**Table 3:** Three-dimensional electroanatomic mapping, ECG and myocardial work.

	SVA	HBP	RVP	SVA vs. HPB *p*	SVA vs. RVP *p*	HBP vs. RVP *p*
RV activation time (ms)	103 (92–140)	104 (95–108)	133 (120–147)	0.969	0.011	0.001
QRS duration (ms)	120 ± 31	123 ± 24	168 ± 23	0.929	0.002	0.002
GLS (%)	−13.5 ± 3.9	−12.5 ± 4.9	−9.8 ± 3.9	0.286	0.004	0.012
GWI (mmHg%)	1027 (855–1763)	1019 (810–2046)	653 (340–978)	0.534	0.003	0.002
GCW (mmHg%)	1648 (1044–2152)	1505 (1138–2200)	1191 (941–1711)	0.075	0.011	0.008
GWW (mmHg%)	217 (125–249)	283 (202–360)	494 (304–674)	0.016	0.004	0.004
GWE (%)	87 (80–90)	82 (73–90)	71 (63–78)	0.049	0.003	0.006
Maximum TTP strain difference (ms)	162 (144–207)	153 (136–237)	184 (153–291)	0.533	0.286	0.530
PSD (ms)	47 (41–69)	50 (35–82)	59 (47–89)	0.878	0.241	0.060

SVA: spontaneous ventricular activation. HBP: His bundle pacing. RVP: right ventricular pacing. RV: right ventricle. GLS: global longitudinal strain. GWI: global work index. GCW: global constructive work. GWW: global wasted work. GWE: global work efficiency. TTP: time to peak. PSD: peak strain dispersion.

**Table 4:** One month follow-up.

	Population *n* = 14
HB lead sensing (mV, *n* = 11)	4.0 (2.5–11.5)
RV lead sensing (mV)	11.1 (5.3–15.3)
HB lead impedance (Ohm, *n* = 11)	427 ± 82
RV lead impedance (Ohm)	560 ± 105
HB lead pacing threshold (at 1.0 ms, V, *n* = 11)	1.5 (0.2–4.6)
RV lead pacing threshold (at 0.4 ms, V)	0.7 (0.4–2.6)

HB: His bundle. RV: right ventricle.

## Data Availability

The data underlying this article will be shared on a reasonable request to the corresponding author.
